# Mimoza: web-based semantic zooming and navigation in metabolic networks

**DOI:** 10.1186/s12918-015-0151-5

**Published:** 2015-02-26

**Authors:** Anna Zhukova, David J Sherman

**Affiliations:** Inria/Université Bordeaux/CNRS joint project-team MAGNOME, 351, cours de la Libération, Talence, F-33405 France

**Keywords:** Metabolic modeling, Visualization, Model generalization

## Abstract

**Background:**

The complexity of genome-scale metabolic models makes them quite difficult for human users to read, since they contain thousands of reactions that must be included for accurate computer simulation. Interestingly, hidden similarities between groups of reactions can be discovered, and generalized to reveal higher-level patterns.

**Results:**

The web-based navigation system Mimoza allows a human expert to explore metabolic network models in a semantically zoomable manner: The most general view represents the compartments of the model; the next view shows the generalized versions of reactions and metabolites in each compartment; and the most detailed view represents the initial network with the generalization-based layout (where similar metabolites and reactions are placed next to each other). It allows a human expert to grasp the general structure of the network and analyze it in a top-down manner

**Conclusions:**

Mimoza can be installed standalone, or used on-line at http://mimoza.bordeaux.inria.fr/, or installed in a Galaxy server for use in workflows. Mimoza views can be embedded in web pages, or downloaded as COMBINE archives.

**Electronic supplementary material:**

The online version of this article (doi:10.1186/s12918-015-0151-5) contains supplementary material, which is available to authorized users.

## Background

Semantic generalization of metabolic network models [1] is a theoretical method designed to aid users in understanding complex models. Generalization identifies and groups into classes biochemically similar metabolites and functionally similar reactions in the network. While we say “similar” in the commonsense way that a biologist would consider that the entities belong to the same class, we mean precisely that the two concepts are related by *is-a* relations in the corresponding ontology. For example, in a generalized model we might group all hexoses, and thus group together most hexose transporters, for a study where the differences between these transporters is not pertinent. Generalization is a kind of dimension reduction in complex models. It can also be used on several models simultaneously: a challenge in comparing models of related organisms, or in reconciling two models of the same organism, is that different curation standards may have been applied to the different models. Generalization can bring disparate models to the same level of abstraction so that they can be compared. To explore the opportunities of model generalization, we implement it here as a practical tool that can be easily adopted and easily integrated into existing workflows.

The *zooming user interface* (ZUI) [2] paradigm has proven to be a powerful tool for representation of data at different scales. It is being adopted for various domains of applications, including cartographic [3], exploratory data visualization [4], collaborative interfaces [5] and biological data [6,7]. The challenge is how to use ZUI-based visualization for semantic generalization of metabolic networks.

### Metabolic network reconstruction and infrastructure

There is a conflict between the level of detail of metabolic models needed for computer simulation and the one that can be easily analyzed by a human curator: Genome-scale metabolic models include thousands of reactions that may participate in organism’s metabolism (e.g., 2 251 reactions in the metabolic network of the bacterium *E. coli* [8], 2 352 reactions in the *yeast 7* metabolic network model of *S. cerevisiae* [9], 7 440 reactions in *recon 2*, a global human metabolism reconstruction [10]), while human experts understand best small-sized networks, containing up to hundreds of nodes [11,12].

**Figure 1:**
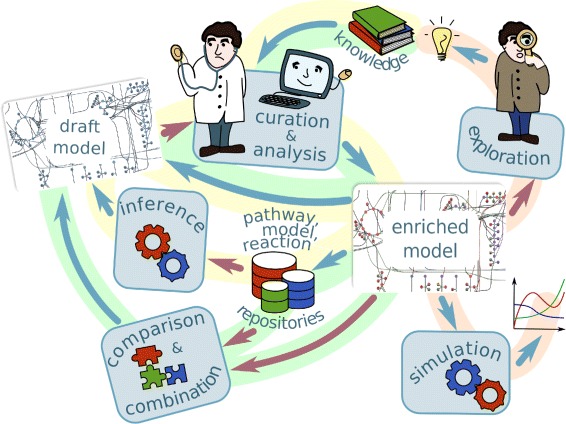
**Metabolic modeling workflow.** The processes highlighted in yellow represent the *model creation cycle*: The draft model is created by model inference tools based on models for similar organism, pathway and reaction information extracted from model repositories and pathway and reaction databases; it is then iteratively improved during the process of curation and analysis. The resulting model can in its turn be added to model repositories. The processes highlighted in red show *model usages*: simulation and knowledge-oriented exploration. The processes highlighted in green describe *comparison and combination of several models*. As the model creation cycle, they also include the curation and analysis stage. The processes represented with the red arrows require human expert’s intervention, and thus need good visualization tools, ways of splitting models into modules and different levels of abstraction.

Metabolic network reconstruction can address various objectives. Examples include creation of a model for a new organism from its genomic data and a reference model for a similar organism; creation of a larger-scale model by combining several models of different aspects of organism’s metabolism; improving an existing model by incorporating new data and new expertise. To accomplish these objectives the following tasks are used (see Figure 1).

#### Inference

The metabolic network reconstruction process is becoming more advanced, and there now exist various tools for semi-automatic model inference, e.g., PathwayTools [13], the RAVEN toolbox [14], KEGGtranslator [15], CoReCo [16], SuBliMinaL [17] (see [18] for a review).

Starting from a model for a related organism or a collection of pathways, and genomic data, they produce a draft model for the target organism. Existing metabolic models can be found in several resources, including Biomodels Database [19], BiGG [20], JWS online [21]. KEGG [22] and Reactome [23,24] provide an extensive collection of pathways.

Models are stored and shared using established formats, such as SBML [25], SBGN-ML [26], CellML [27]. A model represented in these formats can be further enriched with the knowledge from biological databases and ontologies, e.g., ChEBI [28], Uniprot [29], by annotating elements of the models (such as metabolites, reactions) with appropriate identifiers. Further in this manuscript we will consider metabolic models in SBML format.

Although automatic model inference tools and genomic comparison methods are becoming steadily more sophisticated, they may still leave gaps in the model or add erroneous reactions. The intrinsic and extrinsic correctness of the model should be checked during the phases of analysis and curation.

#### Curation and analysis

The inferred draft network needs to be refined during several iterations of analysis, curation and improvement [17,30]. The goal of the *model analysis* is to verify that the model does not contain inner contradictions and errors, e.g., that the network is connected; the transport reactions between compartments are well defined; the reactions are chemically balanced, etc. Various model analysis tools, e.g, FASTGAPFILL [31] for gap filling, CellNetAnalyser [32] for for finding dead ends and blocked reactions, SuBliMinaL Toolbox [17] for reaction balancing, can facilitate model analysis; but human expert’s knowledge on organism’s metabolism still plays an important role.

*Curation* is performed to ensure, first, that all of the knowledge that the experts deem pertinent is recorded in the model, and second, that the knowledge is recorded in a coherent way. The first depends on the requirements of the experts: a model for a cell factory used in an industrial process would need precise kinetics but may only require the reactions active in steady state that participate in the pathway that produces or consumes the target molecule, whereas a whole-genome model used to understand functional dependencies between genes would need to be as complete as possible but may not require reaction kinetics. The second concerns the internal consistency of what is recorded: metabolites and reactions must be annotated with ontology terms from appropriate knowledge bases,

reaction stoichiometry must be consistent, transport between compartments must be assured, and so on. Curation and analysis of models is an iterative process, ideally repeated many times to refine the draft model until the needed level of quality is achieved.

The curation by a human expert requires a means of splitting genome-scale models into smaller units that can be checked and analyzed independently. At a higher level, appropriate levels of abstraction need to be found to allow experts to compare whole genome networks. Good model visualization tools are also required.

#### Simulation

The improved model, created during the iterations of curation and analysis, can be used for computer simulation to obtain numerical results (see [33] for a review of simulation and flux analysis tools). We do not exploit simulation in this manuscript.

#### Exploration

The model can also be used for knowledge-oriented exploration to obtain new knowledge about the processes happening in the organisms’ metabolism, and the relationships between them, e.g., the “redundancy” of the model: discovery of similar reactions, and alternative pathways. Means of splitting genome-scale models into smaller units, appropriate levels of abstraction and good model visualization tools are as important for model exploration task as they are for curation.

#### Comparison and combination

Model comparison and combination is another important task. Possible scenarios include comparison to a different model of the same organism, with potential merging into a new, more complete, model; comparison of a model of a healthy organism to the one of a metabolism suffering from a disease to discover disease-specific metabolic adaptations. A genome-scale model can be created by combining several smaller models, describing different metabolic processes in a species [34], where model comparison is needed to detect overlaps. Such a model can be used as a draft model, and will need to undergo the analysis and curation phase. Finally, a group of models for related species can be compared and combined to produce a concise representation of their common metabolism, to study the common properties of a group, as well as the organism-specific adaptations.

There exist various software facilitating model merging, e.g., semanticSBML [35], OREMPdb [36], PathCase-SB Model Composition Tool [37], but all of them require human expert’s intervention in cases when the models to be merged are incompatible or contradict to each other, as well as for better discovery of common parts. Thereby, after the creation, the combined model becomes a draft and should in its turn undergo the analysis and curation cycle.

By combining these modeling tasks into workflows, as in Figure 1, one can accomplish the modeling objectives listed above.

At least three of the aforementioned tasks (curation, exploration, comparison) require the intervention of a human expert, and thus require methods of dealing with the complexity of the models, e.g., by splitting them into smaller modules, by defining different levels of abstraction, and by visualization.

### Existing visualization approaches

There exist various modeling tools for metabolic networks that also support visualization. Desktop tools include CellDesigner [38], VANTED [39], and Cytoscape [40]. They produce reasonably good visualizations of small networks (up to hundreds of reactions), but become cluttered at the genome-scale level, making the visualization unreadable.

Web-based tools allowing for metabolic network visualization are also available. JWS online [21], for example, provides a mechanism for network visualization using a force-directed layout algorithm [41,42]. It also encounters the aforementioned issues and thus is not capable of providing a readable representation for large networks.

MetDraw [43] is an online tool for genome-scale metabolic model visualization, that makes use of decomposition of the model into compartments and pathways (if the pathway information is present in the model as a *subsystem* annotation of reactions) and duplication of minor metabolites. Metabolite duplication reduces clutter, but the huge number of reactions in the compartments of some models and missing *subsystem* annotations, makes the visualization consume too much space and do not allow a user to grasp the essential structure of the network.

Due to the huge numbers of reactions and of metabolites participating in multiple reactions, we have an uncomfortable choice between either many edge crossings in an automatic visualization of a genome-scale network, or over-duplication of various metabolites making the essential parts of the network disconnected and the visualization too large to grasp. Therefore an approach different to a simple graph layout algorithm is necessary. ZUIs, which can change the size and nature of the content displayed at different zoom levels, provide a pertinent alternative. Two main types of magnification can be considered: *geometric zooming*, in which a region of the network is enlarged; and *semantic zooming*, in which additional properties are introduced with enlargement [7].

Semantic zooming was first introduced for biological data visualization in 1988 with Zomit [6], a generic application programming interface for developing servers for zoomable navigation and visualization, and illustrated with an example of ZoomMap, a prototype browser for HuGeMap human genome database [44]. The work by Jianlu and Laidlaw [45] evaluates geometric zooming with the Google Maps interface on five examples (a gene co-regulation visualization, a gene expression heatmap viewer, a genome browser, a protein interaction network, and neural projections), and describes a positive feedback provided by both domain experts and less experienced users. Another example of a Google Maps-based ZUI is X:map [46], a genome annotation database that supports zoomable data browsing. It does not use semantic zooming, but allows for showing/hiding layers with additional information (EST and GenScan predictions).

There exist several web-based tools that include a zoomable representation of metabolic networks. Genome Projector [47] is a zoomable genome map with multiple views, including a pathway map. The pathway map is based on the Roche Biochemical Pathway wall chart available from the ExPASy proteomics server [48]. The Roche Biochemical Pathway wall chart has a large size and shows the collection of biochemically known molecules, enzymes and reactions. Genome Projector provides a geometric zooming on the map and overlay layers to highlight reactions present in the organism of interest. The list of organisms is fixed to 320 bacterial genomes. The full Roche Biochemical Pathway map with the imposed layout is always shown, but only the reactions of interest (corresponding to the chosen organism) are highlighted.

NaviCell [49] is a web environment that permits exploiting large maps of molecular interactions, including metabolic maps. It allows users to create their own maps, but does not provide a solution to the problem of huge network layout. The map creation is not fully automatic: The user must create a map in CellDesigner, export it as an image and partly manually edit it in a graphical designer to produce intermediate views (possibly with different level of details for semantic zooming). In addition, NaviCell permits a user to split the map into submaps called modules.

Another web-based tool, the Cellular Overview [50] creates interactive diagrams for metabolic maps of organisms in the BioCyc database [51]. It is pathway-oriented, and supports only geometric zooming. Another drawback is that it does not show the compartmentalization.

The Reactome pathway database [23,24] browser provides a zommable visualization of manually curated pathways for 19 organisms. It has two semantic zoom levels: a general representation of organism’s pathways (nodes represent pathways, the edges connect the related ones); and submaps showing the details of each of the pathways, including compartmentalization. Several levels of geometric zoom are available on both semantic zoom levels. Reactome is pathway-oriented. Inside each pathway the layout is imposed: reactions, metabolites, and compartments common to two organisms have the same layout in corresponding representations. On the other hand, the positions and sizes of compartments might differ between pathways of the same organism.

None of the ZUI tools for metabolic map representation described above, except for NaviCell, allow users to input their own models. Moreover, as these examples show, not only geometric zoom but also model decomposition and semantic zoom are important for multi-level visualization of huge models. At the general level, the network needs to be decomposed into several meaningful modules (such as compartments, pathways). If after such a decomposition the model remains complicated (e.g., the mitochondrial compartment of the yeast consensus model [52] containing 230 reactions), a further decomposition is required. We address these issues below by combining model generalization with a ZUI.

## Implementation

### Choosing zoom levels

We address the problem of large-scale metabolic model visualization by combining meaningful decomposition into modules with automatic multi-level abstraction. Decomposition is performed in the following way: The network is first split into compartments; then the model generalization method is applied to each compartment to detect the generalized modules. Thereby, the most appropriate is to adopt 3 levels of semantic zooming:

1. The most abstract level represents compartmentalization of the network, and focuses on such questions as: Are all the compartments present? Are they well connected by transport reactions?
  This level shows the compartments of the model, the transport reactions between them, and other reactions happening inside the cytoplasm. If the model does not describe compartments, this level will be missing.
2. The second level shows the modules inside each of the compartments. The questions that can be addressed at this level include: Are all the reactions or more generally pathways desired by the curators present? are the input-output relations of functional modules consistent with what the expert expects from her knowledge? Does the model show organism-specific adaptations, seen in the model as shortcuts or meanders?
  We use our knowledge-based generalization method to identify the modules inside the compartments. It detects similar metabolites and reactions and clusters them together to represent them as generalized metabolites and reactions with the same structure (numbers of consumed and produced metabolites). The generalized representation reveals the overall structure of the network while hiding the details.
  If no similar metabolites/reactions can be detected by the generalization method (due to the model structure or to missing ChEBI metabolite annotations), this level will be missing.
3. The most detailed level is intended for computer simulation and represents the inner structure of each of the modules with all the metabolites, reactions and their kinetics, stoichiometries and constraints.
  Our method places similar metabolites and reactions (detected at level 2) next to each other, thus simplifying the analysis of their presence.

**Figure 2:**
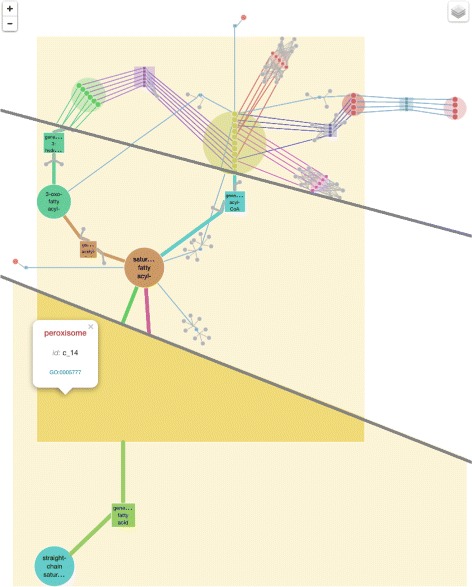
**Three zoom levels.** The most general zoom level (bottom) shows the peroxisome and a generalized transport reaction. The intermediate zoom (middle) shows the generalized processes inside the peroxisome compartment. The most detailed view (top) reveals the metabolites and reactions of the initial model.

Figure 2 shows such a 3-level representation on the example of the model of *β*-oxidation of fatty acids [53] in the peroxisome compartment of a yeast *Y. lipolytica*. The first level (bottom) shows the peroxisome compartment, and the transport reactions; the second level (middle) shows the generalized structure of the peroxisome; the most detailed level (top) represents the complete model, placing semantically similar metabolites and reactions next to each other.

### Model generalization

The metabolic model generalization method [1], which we recall here, groups similar metabolites and reactions in the network based on its structure and the knowledge extracted from metabolite ontologies. A generalization is made specifically for a given model and is maximal with respect to the relations in the model; it respects semantic constraints such as reaction stoichiometry, connectivity, and transport between compartments; and it is performed through a heuristic method that is efficient in practice for genome-scale models. The reader is referred to [1] for these technical details, which are beyond the scope of this article.

To make metabolite grouping semantically meaningful, an ontology describing hierarchical relationships between biochemical entities is used. Each metabolite can be generalized up to one of its ancestors in the ontology. We use the *ChEBI* ontology, as it is the *de facto* standard for metabolite annotation in metabolic networks. If a ChEBI annotation for a metabolite is not present in the model, the method attempts to automatically deduce it by comparing metabolite’s name to ChEBI terms’ names and synonyms.

Reactions that share the same generalized reactants and the same generalized products, are considered equivalent and are factored together into a generalized reaction.

The appropriate level of abstraction for metabolites and reactions is defined by the network itself as the most general one that satisfies two restrictions:

1. *Stoichiometry preserving restriction*: metabolites that participate in the same reaction cannot be grouped together;
2. *Metabolite diversity restriction*: metabolites that do not participate in any pair of similar reactions are not grouped together (as there is no evidence of their similarity in the network).

Overall, the generalization method is composed of three modules:

1. *Aggressive reaction grouping* based on the most general metabolite grouping (defined by ChEBI), in order to generate reaction grouping candidates;
2. *Ungrouping of some metabolites and reactions* to correct for violation of the stoichiometry preserving restriction;
3. *Ungrouping of some metabolites* (while keeping the reaction grouping intact) to correct for violation of the metabolite diversity restriction.

For instance, *(S)-3-hydroxydecanoyl-CoA*, *(S)-3-hydroxylauroyl-CoA* and *(S)-3-hydroxytetradecanoyl-CoA* have a common ancestor *hydroxy fatty acyl-CoA* in *ChEBI*. They can be grouped and generalized into *hydroxy fatty acyl-CoA*, if in the network there is no reaction whose stoichiometry would be changed by such a generalization (stoichiometry preserving restriction), and exist similar reactions that consume or produce them (metabolite diversity restriction).

**Figure 3:**
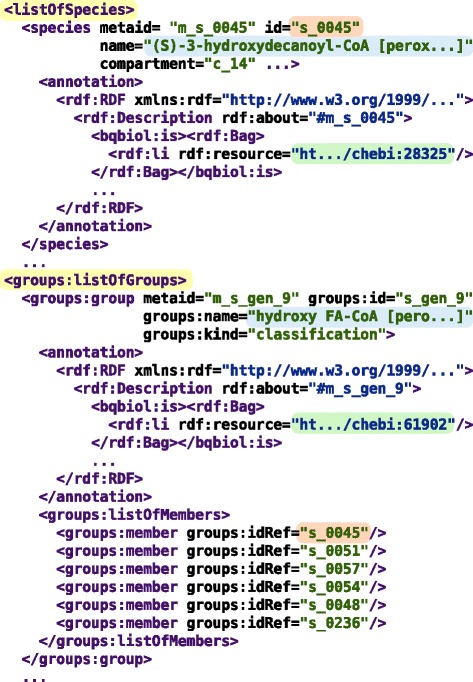
**Representation of a generalized model in SBML level 3 version 1 format with groups extension.** The output SBML file contains the initial model (including the lists of metabolites (called *species* in SBML), reactions, etc.) plus the *listOfGroups* section that represents non-trivial quotient metabolite and reaction sets. In the figure, a group representing a quotient metabolite set of *hydroxy fatty acyl-CoAs* is shown; it includes *(S)-3-hydroxydecanoyl-CoA* (s_0045), *(S)-3-hydroxylauroyl-CoA* (s_0051), etc. Each of those metabolites was previously declared in the *listOfSpecies* section.

The method is available as a python library [54] that operates on models in *SBML* [55] format. It takes an SBML file of level 2 or 3 (any version) and produces an SBML level 3 version 1 file with groups extension [56] that contains the initial model plus groups for all non-trivial similar metabolite and reaction sets (see Figure 3).

The compression that can be achieved with the model generalization method depends on the model structure and on how well the model is annotated with the ChEBI ontology (as the metabolites lacking ChEBI annotations are not generalized). Additional file 1: Table S1 shows the results of the application of the model generalization method to 269 metabolic models from Path2Model project [57]. All those models are genome-scale, the average number of reactions per model is 2 879. The average compression ratio *r* is 1.14:

(1) $$ r = \frac{\text{number of reactions in the initial model}}{\text{number of reactions in the generalized model}}  $$

### Layers layout

To visualize a metabolic network we first represent it as a bipartite graph [58] with two disjoint sets of nodes (metabolites and reactions), and edges that connect the reactions to their substrate and product metabolites. To achieve such a representation, we implemented a converter from SBML to TLP format, that is used by the Tulip graph visualization tool [59]. TLP format stores nodes and edges of the graph, and associates each node and edge to a list of named attributes: standard ones, such as shape, size, color; and user-defined ones, such as, in our case, element type (compartment, reaction or metabolite), ChEBI identifier, group number, gene association, etc. The SBML-to-TLP converter is implemented in python, using libSBML library [60], and is available as a part of Mimoza software.

While layout of large graphs is widely studied [61], the correspondence between the layouts of different semantic zoom levels remains a hard task. To compute the layout for different semantic zoom levels we combine two different approaches.

#### Generalized model layout

In order to lay out the sub-networks corresponding to each of the compartments after the generalization, we use a combination of standard layout algorithms provided by Tulip. We divide the compartment graph into connected components (i.e., subgraphs in which any two nodes are connected to each other by undirected paths, and which are not connected to any additional nodes in the supergraph), using a method provided by Tulip. We then apply an appropriate layout algorithm on each of them. The results are combined together using the *Connected Component Packing* algorithm (provided by Tulip), which places the components close to each other while removing the overlaps between them.

Regarding each of the connected component subgraphs as a directed graph (the direction of the edges is defined by the direction of the corresponding reactions; for reversible reactions edges in both directions are considered), we detect their strongly connected components (i.e., subgraphs where every vertex is reachable from every other vertex) using path-based depth-first search algorithm [62]. Depending on the number of cycles in each strongly connected component subgraph, we choose one of the following layout algorithms, provided by Tulip:

- *Circular Layout* for the strongly connected components with less than 20 cycles (*Circular (OGDF)* [42], with *O*(|*E*|^2^) time and space complexity);
- For components with more cycles we use *Force-Directed Layout* (*FM*^3^*(OGDF)* [63], that has the asymptotic worst-case running time of *O*(|*V*|*log*|*V*|+|*E*|) with linear memory requirements) to reduce the number of edge intersections.

We then represent each strongly connected component as a meta-node [59], apply a *Hierarchical Layout* (*Sugiyama (OGDF)* [64] algorithm (complexity of *O*(|*V*||*E*|) in time and of *O*(|*V*|+|*E*|) in space) on the initial connected component subgraph (that now contains no cycles), and then open the meta-nodes.

To avoid clutter we duplicate all the *minor* metabolites (*oxygen*, *hydrogen*, *water*, *ATP*, etc.) before applying the layout algorithms, so that there is a copy of a minor metabolite for each reaction in which it is used. We then extract a subgraph, containing all but the minor metabolites, apply the combined layout on it, and then place the minor metabolites next to the reactions in which they participate.

#### Generalization-based full model layout

The layout for the full model is based on the corresponding generalized model’s layout. To allow zooming into the generalized model, we keep the same coordinates as in the generalized model for the minor metabolites and the ungeneralized metabolites and reactions, and place similar metabolites or reactions next to each other inside the space used by the corresponding generalized metabolites or reactions in the generalized model.

An edge in the generalized view might expand into several edges in the full-model view, for example, if it is a generalized edge connecting a generalized metabolite to a generalized reaction. The positions of the edges after such an expansion might slightly differ from the corresponding generalized one.

#### Node colors

A different color is assigned to each generalized metabolite/reaction; and is propagated to the corresponding metabolites/reactions of the full model. Minor metabolites are colored grey. Mimoza’s interface includes a checkbox that permits to hide/show minor metabolites.

#### Node sizes

The size of the nodes depends on their nature: minor metabolites are smaller than the other ones; a radius of a generalized metabolite/reaction is calculated as a sum of radiuses of the elements that it groups; compartment sizes are defined by the layouts of the elements inside them, so that the compartments are represented as minimal rectangles containing all the corresponding elements. All major specific (i.e., not generalized) metabolites are of the same size; as well as all specific reactions.

#### Relative positions of compartments

Metabolic models may include several compartments, nested into each other. For example, the *peroxisome* compartment is surrounded by its *membrane*, and contained in *cytoplasm*; the *cytoplasm* is part of the *cell*, which is surrounded by the *cell envelope*.

SBML allows to represent relative positions of the compartments in the model with an optional *outside* tag. However, it is not available in all SBML levels, nor is widely used.

To be able to visualize the compartments correctly even for the SBML models lacking this information, we infer their relative positions from the Gene Ontology (GO) [65]. We associate each compartment with a term from the *cellular component* branch of GO by using annotations in the model if they are present, or matching the compartments’ names otherwise. We then use the *part_of* and *is_a* relationships between the terms in GO to infer relative compartment positions. If no term for a compartment could be found, it is placed on the outer-most level.

#### SBML layout

To store the calculated layout of the model elements we use the layout extension [66] of SBML. It allows to store the coordinates and sizes of the metabolites, reactions and compartments in the model. The TLP-to-SBML layout converter is implemented in python and is available as a part of Mimoza software. If the SBML model submitted by the user contains the layout information, our software uses it for nodes’ positions. Therefore, it is possible to visualize a model with Mimoza, download the resulting SBML with layout annotations, edit it manually or with another software and then revisualize the updated version with Mimoza.

### ZUI

The zoomable interactive representation is achieved using Leaflet [67], a JavaScript library for interactive maps.

**Figure 4:**
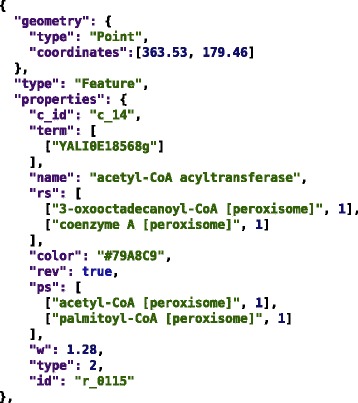
**GeoJSON representation of a reaction.** An SBML reaction is stored as a GeoJSON Point feature, with its layout coordinates encoded in the geometry section. The identifiers, labels and annotations, as well as the information on the reactant and product metabolites are stored as properties. The “type” property value specifies that this GeoJSON feature is a reaction.

We export elements of the network graph (compartments, metabolites and reactions) as map features in GeoJSON format [68] in order to store their coordinates and metadata (e.g., ChEBI annotations for metabolites). Figure 4 shows an example of a reaction represented in GeoJSON format. The TLP-to-GeoJSON converter is implemented in python and is available as a part of Mimoza software.

**Figure 5:**
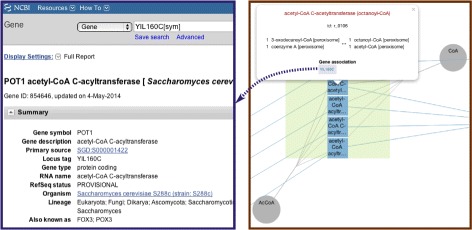
**A reaction pop-up.** (Right part) An example of a pop-up that opens when a user clicks on a reaction: It contains the information on the reaction name, identifier, reactant and product metabolites and their stoichiometries, as well as gene associations. (left part) Gene names are hyperlinks redirecting to the NCBI Gene database [70].

The GeoJSON objects are then added as layers to the map and rendered by Leaflet into clickable elements at corresponding zoom levels. We follow SBGN Process Description language convention [69] to choose the glyphs for model elements’ representation: Metabolites are drawn as circles linked by edges to the reactions where they participate; reactions are represented as squares; compartments are drawn as rectangles. On the semantic zoom levels that show compartments, the corresponding transport reactions are connected to compartments. On the more detailed zoom levels, where the metabolites inside those compartments are shown, these reactions are connected to the corresponding metabolites. When a user clicks on a map element a pop-up appears (see Figure 5) showing its name, identifier and additional information, e.g., gene associations and formulas for reactions. Two overlays allow user to show or hide minor metabolites (e.g., water, oxygen, hydrogen, etc.), and transport reactions.

### Embedding

After the visualization with Mimoza is done, we provide a link for embedding the view in another web page.

### Download and distribution

One can use Mimoza in three different ways:

1. As a standalone application. All Mimoza code is open-source and can be downloaded from the project web page [71] and installed on a local server.
2. On the Mimoza web server. Mimoza web server [71] lets one test visualization for smaller SBML models, with the possibility to download the result as a COMBINE archive [72], including the SBML file with groups (to store the metabolite and reaction groupings) and layout (to store the element coordinates) extensions, GeoJSON files with the coordinates of model elements, and the HTML, CSS and JavaScript files that are needed to view the visualization in a browser.
3. As a Galaxy [73] project tool, so that generation of Mimoza views can be included in a Galaxy workflow. The Galaxy wrapper for Mimoza is available for download from the project web page.

### Pipeline

The overall Mimoza pipeline contains 5 steps:

1. The user submits a model in SBML format (level 2 or 3, any version) via a web form.
2. If the model does not yet contain groups, it is generalized using the model generalization method, and the resulting SBML file (level 3 version 1 with groups extension) is made available to the user.
3. The SBML file with groups of similar metabolites and reactions is converted into a Tulip graph: metabolite nodes are connected by edges to the nodes of the reactions in which they participate. The generalized metabolites and reactions form quotient nodes. The Tulip graph is split into sub-graphs corresponding to different compartments, and layout algorithms are applied to them.
4. The compartment sub-graphs are exported in GeoJSON format and rendered by the Leaflet library into an interactive map that is represented to the user.
5. The result can either be browsed on the Mimoza web page directly, or downloaded as a COMBINE archive and embedded into a different website.

## Results and discussion

To illustrate the use of Mimoza and compare it with other available ZUI tools, we visualized the yeast consensus genome-scale metabolic network model [52]. The result can be found at http://mimoza.bordeaux.inria.fr/yeast4. Mimoza automatically split the network into compartments and created a 3-level visualization for each of them.

We visualized the same model using MetDraw with no manual adjustments. The resulting SVG file^a^ has only one zoom level with lots of clutter, that does not allow one to see the structure of the network.

Cellular Overview does not allow one to visualize a model provided by a user, but has a map of metabolism of *Saccharomyces cerevisiae*.^b^ It has a clear non-overlapping representation of various pathways present in the model, but does not show the compartmentalization. It is not automatic and is pathway-oriented, thus is not suitable for models having no pathway metadata. The zoom-in shows additional labels but all the metabolites and reactions are present at all the levels, making the elements at the most general level very small and hard to analyze.

NaviCell does not allow to visualize an SBML model automatically. Genome Projector only contains maps for bacterial genomes and does not permit user’s model input.

Neither Reactome allows users to visualize their own models, but it contains a pathway map for *Saccharomyces cerevisiae*.^c^ It has two semantic zoom levels: a visualization of a list of pathways present in the model, and submaps corresponding to each of them. The representation of each pathways is very clear, and has several geometric zoom levels. However, it is not always space-efficient as it contains gaps due to reactions present in other organisms but absent in *S. cerevisiae*. Another particularity is that while the positions of elements common to different organisms are conserved within a pathway, their positions might differ between different pathways of the same organism. In Mimoza, on the contrary, the positions of the reactions and metabolites are conserved between the compartments of the same organisms; but the layout of common processes (e.g., pathways) in different organisms’ visualizations might differ in the current implementation.

Table 1 summarizes the comparison of Mimoza to other ZUI tools. Mimoza especially targets draft models during curation, allowing one to visualize them fully automatically and helps to analyze them in a top-down manner, starting from the general structure and going down to the details. The generalized level differentiates it from other tools, since it shows both the overall network structure and fine-grain visualization in the most detailed level, automatically placing semantically similar metabolites next to each other. Mimoza does not depend on pathway information, automatically infers the relative compartment placement (e.g., places organelles inside the cytoplasm) and exploits a model in SBML format with ChEBI annotations for metabolites (if no annotations are present, it tries to infer them automatically based on metabolites’ names).

**Table 1:** **Comparison of ZUIs for metabolic models**

		**Semantic**	**User’s**	**Automatic**	
**Tool name**	**Imposed layout**	**Zoom**	**Model**	**Layout**	**Modules**
Genome projector	yes	no	no	-	no
NaviCell	no	if created	yes	no	yes
		by user			
Cellular overview	yes	no	no	-	no
Reactome	yes (same pathw. diff. org.) /	yes	no	-	yes
	no (diff. pathw. same organism)				
Mimoza	no	yes	yes	yes	yes

Using generalization to compare two metabolic networks makes most sense if they have equivalent generalized nodes that can be placed in corresponding positions in the two layouts. Mimoza currently handles this correspondence between zoom levels of the same network, but does not guarantee such correspondence when two networks are laid out independently. To meet this challenge, three strategies can be explored. The first is to use constrained layout [74], to impose the positions of key features in one network on the corresponding features of the second network. The second is also to use constrained layout, with a catalog of standard positions for common motifs in generalized maps; for example, always lay out the generalized *β*-oxidation of fatty acids as a 4-step cycle, with standard positions for the generalized metabolites common for all the networks that incorporate *β*-oxidation. The third strategy, which we are in the process of testing, is to learn a common layout by generalizing the union of the two networks. The idea is to combine the reactions into one set, run the generalization procedure on the union to fix the positions of the common features, then to build each of the layouts using only its own set of nodes. Each network layout only contains its own nodes, but the common nodes of the two networks will be in common positions.

Finally, the API of the Leaflet framework used for the interactive navigation can be used to integrate the maps with other web-based tools, such as annotation editors or simulation software.

Mimoza is currently targeted to metabolic networks. While it can provide a geometric zooming visualization of a generic SBML model (e.g., a signaling network), the knowledge-based generalization, and therefore semantic zooming, depends on the ChEBI ontology and is intended for metabolic models. A domain-specific adaptation of the generalization method (e.g., use of a domain-specific ontology instead of ChEBI, that is targeted to metabolism) might allow Mimoza to assist in modeling of other kinds of biological networks.

## Conclusions

We have implemented Mimoza, a novel software tool for automatically constructing zooming user interfaces for genome-scale metabolic models. By exploiting *model generalization*, Mimoza reduces the dimension of the model’s network at outer zoom levels, and intelligently co-localizes equivalent reactions and molecular species at inner zoom levels. Consequently the biological user may efficiently navigate the high-level structure of the model; whether the goal is to understand the model or to search for errors, Mimoza exposes the important features at out zoom levels and and hides the specific details in the inner ones. We provide an efficient, useful tool that is easy to adopt and, through the use of standards such as SBML and the ChEBI ontology, is easy to integrate into existing expert-centered modeling pipelines. By carefully combining model generalization with adaptive layout and open-source cartographic software, the Mimoza web server requires just a browser with Javascript. Mimoza is open source and can also be installed locally, as described on the web page, and depends on libSBML, Tulip, and Python.

## Availability and requirements

**Project name:** Mimoza**Project home page:**http://mimoza.bordeaux.inria.fr**Operating system(s):** Platform independent**Programming language:** Python, JavaScript**Other requirements:** JavaScript should be enabled in the web browser. The standalone Mimoza application requires Python 2.7; libSBML-experimental ≥ 5.9 for Python with groups and layout extensions; Leaflet 0.7.3; jQuery 2.1.1 and jQuery-ui 1.10.4; Tulip ≥ 4.0 for python; and model generalization library^d^.**License:** CeCILL (GPL compatible)**Any restrictions to use by non-academics:** no restrictions

## Endnotes

^a^ MetDraw – http://www.metdraw.com/metdraw/bc7df60221ba314c383b1bf6e7dad4c3056f92bb.

^b^ Cellular Overview – http://biocyc.org/overviewsWeb/celOv.shtml.

^c^ Reactome – http://www.reactome.org/PathwayBrowser/\#SPECIES=68322\&DIAGRAM=5686439.

^d^ Model Generalization – http://metamogen.gforge.inria.fr.

## Additional file

Additional file 1
**Table S1.** Performance of the model generalization method.
