# Supplementary material for: Mimoza: web-based semantic zooming and navigation in metabolic networks
Source: BMC Syst Biol. 2015 Feb 26;9:10. doi: 10.1186/s12918-015-0151-5 (PMC4345040; doi:10.1186/s12918-015-0151-5)
Supplement: Additional file 1 — Table S1. Performance of the model generalization method. [file 12918_2015_151_MOESM1_ESM.pdf]

Additional file 1

Table S1 Performance of the model generalization method

Model	Number of reactions (initial model)	Number of reactions (generalized model)	Compression rate
BMID000000140205	4010	3469	1.16
BMID000000140206	3631	3180	1.14
BMID000000140207	889	801	1.11
BMID000000140208	2366	1989	1.19
BMID000000140209	4088	3576	1.14
BMID000000140210	4597	3985	1.15
BMID000000140211	2868	2538	1.13
BMID000000140212	3887	3367	1.15
BMID000000140213	3824	3329	1.15
BMID000000140214	2678	2447	1.09
BMID000000140215	1280	1147	1.12
BMID000000140216	3319	2924	1.14
BMID000000140217	3651	3204	1.14
BMID000000140218	2209	1890	1.17
BMID000000140219	2768	2279	1.21
BMID000000140220	4162	3665	1.14
BMID000000140221	2027	1921	1.06
BMID000000140222	2550	2155	1.18
BMID000000140223	2970	2632	1.13
BMID000000140224	1723	1525	1.13
BMID000000140225	2271	1944	1.17
BMID000000140226	3108	2794	1.11
BMID000000140227	4635	3955	1.17
BMID000000140228	1582	1404	1.13
BMID000000140229	3023	2670	1.13
BMID000000140230	2192	1932	1.13
BMID000000140231	968	891	1.09
BMID000000140232	371	328	1.13
BMID000000140233	3856	3306	1.17
BMID000000140234	2527	2158	1.17
BMID000000140235	1840	1589	1.16
BMID000000140236	3555	3095	1.15
BMID000000140237	1365	1228	1.11
BMID000000140238	3960	3476	1.14
BMID000000140239	2588	2197	1.18
BMID000000140240	659	593	1.11
BMID000000140241	3168	2763	1.15
BMID000000140242	3203	2835	1.13
BMID000000140243	3893	3425	1.14
BMID000000140244	4325	3785	1.14
BMID000000140245	4387	3858	1.14
BMID000000140246	4437	3837	1.16
BMID000000140247	4506	3917	1.15

BMID000000140248	4156	3612	1.15
BMID000000140249	1993	1797	1.11
BMID000000140250	2213	1933	1.14
BMID000000140251	3034	2733	1.11
BMID000000140252	3374	2953	1.14
BMID000000140253	2469	2069	1.19
BMID000000140254	1326	1196	1.11
BMID000000140255	3956	3430	1.15
BMID000000140256	2665	2378	1.12
BMID000000140257	3363	2823	1.19
BMID000000140258	2913	2513	1.16
BMID000000140259	2008	1784	1.13
BMID000000140260	4608	3983	1.16
BMID000000140261	1293	1187	1.09
BMID000000140262	3064	2696	1.14
BMID000000140263	3705	3260	1.14
BMID000000140264	2435	2051	1.19
BMID000000140265	2281	2066	1.10
BMID000000140266	2623	2398	1.09
BMID000000140267	1811	1598	1.13
BMID000000140268	4321	3707	1.17
BMID000000140269	3571	2983	1.20
BMID000000140270	2199	1858	1.18
BMID000000140271	3941	3447	1.14
BMID000000140272	2354	2071	1.14
BMID000000140273	2010	1789	1.12
BMID000000140274	1960	1810	1.08
BMID000000140275	3277	2945	1.11
BMID000000140276	3333	2978	1.12
BMID000000140277	4625	4030	1.15
BMID000000140278	987	933	1.06
BMID000000140279	4060	3641	1.12
BMID000000140280	2090	1837	1.14
BMID000000140281	2474	2294	1.08
BMID000000140282	1667	1479	1.13
BMID000000140283	1680	1504	1.12
BMID000000140284	3887	3388	1.15
BMID000000140285	3376	2894	1.17
BMID000000140286	2752	2429	1.13
BMID000000140287	4095	3580	1.14
BMID000000140288	3799	3266	1.16
BMID000000140289	4336	3676	1.18
BMID000000140290	2041	1774	1.15
BMID000000140291	4089	3578	1.14
BMID000000140292	3482	2922	1.19
BMID000000140293	3836	3392	1.13
BMID000000140294	3880	3381	1.15
BMID000000140295	1481	1339	1.11
BMID000000140296	3107	2783	1.12
BMID000000140297	3799	3318	1.14
BMID000000140298	2358	2102	1.12
BMID000000140299	1963	1700	1.15
BMID000000140300	2796	2512	1.11
BMID000000140301	1203	1110	1.08
BMID000000140302	406	366	1.11

BMID000000140303	3145	2748	1.14
BMID000000140304	3740	3289	1.14
BMID000000140305	1640	1502	1.09
BMID000000140306	2058	1839	1.12
BMID000000140307	2732	2475	1.10
BMID000000140308	1648	1459	1.13
BMID000000140309	1168	1082	1.08
BMID000000140310	3888	3429	1.13
BMID000000140311	1673	1534	1.09
BMID000000140312	2826	2469	1.14
BMID000000140313	5056	4428	1.14
BMID000000140314	1425	1319	1.08
BMID000000140315	1116	1036	1.08
BMID000000140316	2138	1950	1.10
BMID000000140317	3535	2972	1.19
BMID000000140318	1519	1363	1.11
BMID000000140319	2117	1927	1.10
BMID000000140320	2531	2269	1.12
BMID000000140321	3513	3071	1.14
BMID000000140322	4339	3716	1.17
BMID000000140323	597	541	1.10
BMID000000140324	1245	1156	1.08
BMID000000140325	2513	2334	1.08
BMID000000140326	2607	2399	1.09
BMID000000140327	2244	1930	1.16
BMID000000140328	974	872	1.12
BMID000000140329	3231	2880	1.12
BMID000000140330	2011	1828	1.10
BMID000000140331	1693	1542	1.10
BMID000000140332	4269	3669	1.16
BMID000000140333	1633	1509	1.08
BMID000000140334	3546	3107	1.14
BMID000000140335	1650	1516	1.09
BMID000000140336	1928	1771	1.09
BMID000000140337	4316	3703	1.17
BMID000000140338	1548	1377	1.12
BMID000000140339	1879	1697	1.11
BMID000000140340	656	635	1.03
BMID000000140341	2302	1937	1.19
BMID000000140342	3103	2699	1.15
BMID000000140343	2655	2402	1.11
BMID000000140344	1787	1687	1.06
BMID000000140345	3189	2682	1.19
BMID000000140346	1921	1778	1.08
BMID000000140347	2999	2675	1.12
BMID000000140348	1930	1818	1.06
BMID000000140349	2895	2627	1.10
BMID000000140350	1799	1545	1.16
BMID000000140351	3620	3170	1.14
BMID000000140352	2586	2351	1.10
BMID000000140353	2927	2631	1.11

BMID000000140354	4744	4174	1.14
BMID000000140355	4673	4017	1.16
BMID000000140356	4670	4043	1.16
BMID000000140357	1673	1485	1.13
BMID000000140358	4382	3794	1.15
BMID000000140359	3200	2852	1.12
BMID000000140360	807	767	1.05
BMID000000140361	3400	2970	1.14
BMID000000140362	5819	4948	1.18
BMID000000140363	1311	1166	1.12
BMID000000140364	3185	2785	1.14
BMID000000140365	3962	3454	1.15
BMID000000140366	4107	3571	1.15
BMID000000140367	3490	3092	1.13
BMID000000140368	1738	1628	1.07
BMID000000140369	2317	2004	1.16
BMID000000140370	4068	3565	1.14
BMID000000140371	4272	3782	1.13
BMID000000140372	2109	1834	1.15
BMID000000140373	1259	1137	1.11
BMID000000140374	2952	2547	1.16
BMID000000140375	944	892	1.06
BMID000000140376	828	788	1.05
BMID000000140377	2595	2370	1.09
BMID000000140378	4528	3864	1.17
BMID000000140379	4014	3519	1.14
BMID000000140380	1609	1484	1.08
BMID000000140381	4379	3779	1.16
BMID000000140382	1769	1563	1.13
BMID000000140383	2365	2009	1.18
BMID000000140384	3926	3477	1.13
BMID000000140385	3510	3099	1.13
BMID000000140386	4133	3579	1.15
BMID000000140387	3096	2779	1.11
BMID000000140388	2010	1791	1.12
BMID000000140389	3635	3187	1.14
BMID000000140390	2416	2150	1.12
BMID000000140391	2861	2535	1.13
BMID000000140392	3013	2708	1.11
BMID000000140393	1659	1463	1.13
BMID000000140394	3147	2770	1.14
BMID000000140395	3317	2908	1.14
BMID000000140396	2958	2649	1.12
BMID000000140397	2022	1792	1.13
BMID000000140398	2715	2417	1.12
BMID000000140399	2589	2203	1.18
BMID000000140400	2765	2445	1.13
BMID000000140401	3418	3017	1.13
BMID000000140402	2979	2680	1.11
BMID000000140403	3301	2907	1.14
BMID000000140404	3586	3000	1.20
BMID000000140405	1935	1820	1.06
BMID000000140406	2768	2448	1.13

BMID000000140407	2771	2484	1.12
BMID000000140408	4011	3375	1.19
BMID000000140409	3853	3397	1.13
BMID000000140410	2787	2531	1.10
BMID000000140411	3029	2651	1.14
BMID000000140412	4639	3967	1.17
BMID000000140413	1939	1668	1.16
BMID000000140414	2805	2528	1.11
BMID000000140415	1289	1181	1.09
BMID000000140416	1608	1422	1.13
BMID000000140417	3099	2768	1.12
BMID000000140418	2859	2603	1.10
BMID000000140419	2059	1787	1.15
BMID000000140420	3833	3330	1.15
BMID000000140421	3042	2756	1.10
BMID000000140422	2131	1843	1.16
BMID000000140423	4512	3900	1.16
BMID000000140424	1711	1545	1.11
BMID000000140425	3729	3235	1.15
BMID000000140426	1176	1086	1.08
BMID000000140427	2551	2160	1.18
BMID000000140428	2253	1935	1.16
BMID000000140429	2765	2491	1.11
BMID000000140430	3734	3351	1.11
BMID000000140431	1276	1184	1.08
BMID000000140432	3914	3395	1.15
BMID000000140433	2725	2362	1.15
BMID000000140434	4294	3661	1.17
BMID000000140435	4395	3765	1.17
BMID000000140436	2958	2614	1.13
BMID000000140437	2704	2474	1.09
BMID000000140438	3824	3391	1.13
BMID000000140439	2996	2686	1.12
BMID000000140440	2371	2172	1.09
BMID000000140441	1848	1743	1.06
BMID000000140442	2732	2484	1.10
BMID000000140443	2627	2373	1.11
BMID000000140444	3027	2639	1.15
BMID000000140445	4260	3706	1.15
BMID000000140446	3733	3311	1.13
BMID000000140447	4005	3519	1.14
BMID000000140448	2114	1892	1.12
BMID000000140449	4333	3708	1.17
BMID000000140450	4198	3731	1.13
BMID000000140451	3114	2729	1.14
BMID000000140452	4337	3737	1.16
BMID000000140453	2492	2196	1.13
BMID000000140454	5072	4335	1.17
BMID000000140455	4051	3559	1.14
BMID000000140456	2778	2513	1.11
BMID000000140457	1753	1521	1.15
BMID000000140458	3846	3369	1.14
BMID000000140459	2545	2290	1.11
BMID000000140460	4547	4057	1.12

BMID000000140461	3337	2961	1.13
BMID000000140462	389	347	1.12
BMID000000140463	4895	4216	1.16
BMID000000140464	1078	1030	1.05
BMID000000140465	3114	2791	1.12
BMID000000140466	3546	2963	1.20
BMID000000140467	4355	3745	1.16
BMID000000140468	4418	3823	1.16
BMID000000140469	3563	3189	1.12
BMID000000140470	4095	3573	1.15
BMID000000140471	3551	3209	1.11
BMID000000140472	1743	1555	1.12
BMID000000140473	4040	3446	1.17
**Average:**	2879	2532	1.14
